# Transcriptomic basis for an antiserum against *Micrurus corallinus *(coral snake) venom

**DOI:** 10.1186/1471-2164-10-112

**Published:** 2009-03-16

**Authors:** Luciana I Leão, Paulo L Ho, Inacio de LM Junqueira-de-Azevedo

**Affiliations:** 1 Centro de Biotecnologia, Instituto Butantan, Av. Vital Brasil, 1500, 05503-900, São Paulo, SP, Brazil; 2 Departamento de Genética e Biologia Evolutiva, Instituto de Biociências, Universidade de São Paulo, SP, Brazil

## Abstract

**Background:**

*Micrurus corallinus *(coral snake) is a tropical forest snake belonging to the family Elapidae. Its venom shows a high neurotoxicity associated with pre- and post-synaptic toxins, causing diaphragm paralysis, which may result in death. In spite of a relatively small incidence of accidents, serum therapy is crucial for those bitten. However, the adequate production of antiserum is hampered by the difficulty in obtaining sufficient amounts of venom from a small snake with demanding breeding conditions. In order to elucidate the molecular basis of this venom and to uncover possible immunogens for an antiserum, we generated *expressed sequences tags *(ESTs) from its venom glands and analyzed the transcriptomic profile. In addition, their immunogenicity was tested using DNA immunization.

**Results:**

A total of 1438 ESTs were generated and grouped into 611 clusters. Toxin transcripts represented 46% of the total ESTs. The two main toxin classes consisted of three-finger toxins (3FTx) (24%) and phospholipases A_2 _(PLA_2_s) (15%). However, 8 other classes of toxins were present, including C-type lectins, natriuretic peptide precursors and even high-molecular mass components such as metalloproteases and L-amino acid oxidases. Each class included an assortment of isoforms, some showing evidence of alternative splicing and domain deletions. Five antigenic candidates were selected (four 3FTx and one PLA_2_) and used for a preliminary study of DNA immunization. The immunological response showed that the sera from the immunized animals were able to recognize the recombinant antigens.

**Conclusion:**

Besides an improvement in our knowledge of the composition of coral snake venoms, which are very poorly known when compared to Old World elapids, the expression profile suggests abundant and diversified components that may be used in future antiserum formulation. As recombinant production of venom antigens frequently fails due to complex disulfide arrangements, DNA immunization may be a viable alternative. In fact, the selected candidates provided an initial evidence of the feasibility of this approach, which is less costly and not dependent on the availability of the venom.

## Background

The coral snake (genus *Micrurus*) is the most abundant, diverse and representative member of the family Elapidae in the New World. It has a wide geographic distribution which covers the southwest United States, Central America, and southern Argentina [1]. Compared to the family Viperidae, the numbers of accidents caused by the coral snake are not great. Coral snakes are not aggressive and only attack when threatened. Still, when an accident occurs, the symptoms are usually severe, leading to death by asphyxia after only 5 or 6 hours due to strong neurotoxic effects [2].

In Brazil, cases of envenoming by coral snakes are caused mainly by *Micrurus corallinus *and *Micrurus frontalis*, species inhabiting in highly populated areas in the Central, South and Southeast regions. Many of their characteristics, such as ophiophagous diet, fossorial habit and living in tropical latitudes, make it difficult to obtain and keep them in captivity. This limitation in maintenance, the small size of their venom glands and, consequently, low production of venom have been the major factors hindering the production of Brazilian anti-elapidic serum. Furthermore, the Butantan Institute, in Sao Paulo, utilizes almost all of the venom obtained to produce the anti-elapidic serum, limiting biochemical studies [3]. In fact, the quantity of venom available for the generation of serum is not enough to supply the national needs. While the incidence of accidents is small when compared to that for other genera, the wide geographic dispersion of *Micrurus *and the lethality of its bite require the serum to be distributed all over the country, raising its demand.

Nowadays, the transcriptomic analysis of venom glands to obtain a general profile of the toxins composing the venom is a common experimental approach, applied especially in snakes of the families Viperidae [4-8] and Colubridae [9]. Nevertheless, there are no systematic transcriptome reports of this kind for the family Elapidae, neither from American coral snakes nor from African-Asian species. Moreover, considering the difficulties in obtaining the venom even for antiserum production, a complete set of the most abundant cDNAs from the venom glands of coral snake species could open possibilities for alternative ways of immunization. One of them is the production of recombinant proteins for immunization, which is an obvious choice, since horse hyper-immunization demands large amounts of proteins and requires robust expression systems, such as those using *E. coli*. However, prokaryotic systems fail to express complex disulfide-bonded proteins, as in the case of snake toxins. Several of our attempts to produce *M. corallinus *recombinant toxins involved complicated refolding and eventually resulted in time-consuming procedures [10,11]. A more elegant approach is the DNA immunization with plasmids expressing exogenous DNA in animal cells, thus stimulating immunological responses [12]. For snake toxins, Harrison and colleagues [13] produced high titers of antibodies after DNA immunization using snake toxin cDNAs, showing a potential systemic treatment of injuries caused by Viperidae snake bites. They then immunized mice with a synthetic chimeric DNA containing seven different epitopes from snake venom metalloproteases expressed in the venom glands of the viper *Echis ocellatus *[14].

The dataset of *Micrurus corallinus *transcriptome presented here aims not only to describe the putative toxins expressed by this sinuous Elapidae snake, but also to be a source of potential antigens for an alternative production of antiserum. Therefore, some cDNAs meeting eligible criteria, such as abundance, were selected and injected into mice for a preliminary evaluation of their capacity to produce an immune response as DNA immunogens.

## Results and Discussion

### Transcriptomic analysis

The EST databank of *M. corallinus *contains 1438 sequences grouped into 611 clusters using CAP3 software with 98% identity. Since the three-finger proteins (3FTx) present in *M. corallinus *venom are very diverse and a few substitutions of residues could change the clade in which they are classified, some of the clusters were inspected and manually regrouped to avoid joining different EST products in the same cluster.

The 611 clusters of contiguous sequences include 483 formed by individual clones (*singlets*) and 128 clusters with two or more sequences (*contigs*). After the application of a filter against sequences of ribosomal RNA, mitochondrial DNA and other contaminants, 5% of the clones were removed for the subsequent statistical analysis.

The analysis using Blast revealed that 485 clusters (corresponding to 1250 clones, or 92% of the total) produced significant similarities with the database (e-value < 10^-05^), and that 103 clusters (107 clones or 8% of total) could not be identified. The information obtained was separated into two general functions of venom gland transcripts: toxins (toxic functions) and other transcripts involved in all cellular functions (non-toxins). Toxin transcripts totaled 46% of all clones in 93 clusters. Non-toxins also totaled 46% of all clones but in 392 clusters. Therefore, the non-toxins are less redundant (1.6 clone/cluster) than toxins (6.7 clones/cluster), which means that each toxin is, on average, four times more transcribed than a cellular transcript.

**Figure 1:**
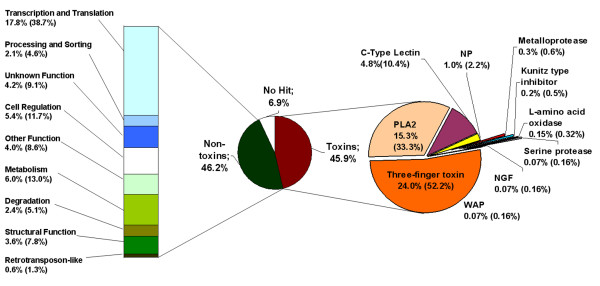
**Relative proportion of each category of product found in *M. corallinus *venom gland transcriptome**. Toxin, Non-toxin and No Hit categories are, respectively, clusters matching GenBank snake toxin sequences, non snake toxin sequences, and any sequence with an e-value < 10e-05 on Blastx search or inconclusive nucleotide matches on Blastn. Non-toxins are categorized by their cellular function, whereas toxins by their structural type. Percentages of total ESTs and of the ESTs in the category (in parentheses) are presented.

Among the 10 particular types of toxins observed (Figure 1), three-finger proteins (3FTx) are the most abundant transcripts, followed by type A_2 _phospholipase (PLA_2_). In smaller numbers are the C-type lectins and the precursor of natriuretic peptide (NP). The other classes of toxins are less abundant. The profile of putative cellular proteins (Figure 1, left) showed a pattern expected from a secretory gland. The most expressed of these transcripts are related to mRNA transcription and protein translation.

### Analysis of the putative toxins

#### Three-Finger Toxins (3FTx)

The 3FTx are the most abundant group of possible toxins, comprising the majority of expressed sequences (~52% of the total toxin clones). These proteins are small, with less than 80 amino acids and generally have four or five disulfide bonds. They have very different pharmacological effects, but are generally associated with post-synaptic blockade.

The analysis of the 3FTx revealed that 326 clones (~24% of total) could be grouped into 55 clusters. Many clusters had to be recreated manually after the initial grouping made by the CAP3 program, because it was noted that some initial contigs were actually grouping different variants. Therefore, each initial contig was inspected to identify the SNPs (*single nucleotide polymorphisms*) common to more than one sequence in the contig. When these common SNPs were found, the quality of their base pairs at each point was evaluated, and the respective sequences were removed and rearranged in a new cluster. It was not possible to determine if a single animal shows all these isoforms or if they are expressed by distinct animals, since the cDNA library was constructed using 10 coral snakes.

**Figure 2:**
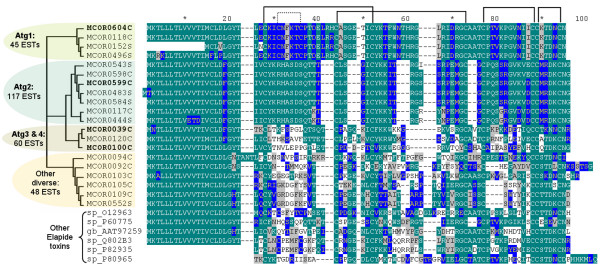
**Alignment of translated 3FTx clusters from *M. corallinus *transcriptome (MCORs) and from other Elapidae species (referred by accession numbers)**. Sequences can be organized in different groups, as emphasized by a Neighbor Joining tree on the left, from which some clusters were selected (bold) to be used in the DNA immunization.

It is not an easy task to classify this kind of toxins based on their sequences, since they are short proteins subjected to high evolutionary pressures, resulting in extreme diversity [15], leaving conserved only the cysteines and some particular features such as the size of the C-terminal region [16]. The many attempts to group them usually resulted in orphan groups that mostly do not reflect their known activities. Besides that, since only few cDNAs are known from *Micrurus*, it is even more complicated to identify the possible functions of the toxins based on orthologs in other species. Therefore, we opted to group these toxins according to the similarity among themselves and their relative abundances (Figure 2) and [see Additional file 1].

Two groups are much more abundant than the rest, representing the majority of the 3FTx (Figure 2). Both contain sequences that are very similar to α-neurotoxin isoforms previously described in *Micrurus corallinus *[3]. The first group, named here Atg1 (selected as antigen 1, as discussed later), was similar to a cDNA called neurotoxin homolog 8 (nxh8), previously isolated by Ho et al. (1995), which is a peculiar form that differs from most 3FTx. After a 21-amino acid signal peptide, the mature form consists of 65 amino acids, along with the possible presence of an extra disulfide bond in the first loop. Most short 3FTx have only four very conserved disulfide bridges but few unrelated toxins show this extra pair of cysteines (Figure 2). The second group, named Atg2 (selected as antigen 2, as discussed later), refers to more typical 3FTx, the neurotoxin homologs 7/3/1 (nxh7, nxh3, nxh1 cDNAs), as previously described [17]. Some of the clusters showed differences in the signal peptide region and some amino acids in the mature protein region. The mature protein consists of 57 amino acids. Comparing these two groups, Atg1 displays just 42% similarity with the Atg2 group. The rest of the 3FTx, some of them also highly transcribed, represent new proteins with an identity value of no more than 50% to the sequences of 3FTx in the databanks (Figure 2).

#### Phospholipase A_2 _(PLA_2_)

With 120 amino acids and 6 or 7 disulfide bonds, PLA_2 _are ubiquitous enzymes in venoms, composed of alpha-helix and some beta-sheet structures [18]. Some PLA_2_s are pre-synaptic neurotoxins or β-neurotoxins with specific targets in neuromuscular junctions [19], promoting an inhibition in the release of neuromediators. Some others act like a myotoxin that depolarizes muscular cell membranes[20], leading to necrosis. PLA_2 _may exert neurotoxic actions by binding to nerve cell membranes and catalyzing phospholipid hydrolysis, with the production of lysophospholipids and free fatty acids [21].

The most abundant cluster obtained was MCOR0036C, with 196 clones. This cluster shows 100% identity with the PLA_2 _sequence of *Micrurus corallinus *from the database (AY157830). This PLA_2 _was expressed in *E. coli *and immunologically characterized [11]. It has, in position 49, one aspartate (Asp49) intimately involved in Ca^2+ ^binding, having an essential function in the catalytic activity of this enzyme. This residue is highly conserved in catalytically functional PLA_2_, but in many Viperidae myotoxic PLA_2_s, it is substituted by a lysine residue (Lys49) [22]. No such latter form was detected in this transcriptome.

**Figure 3:**
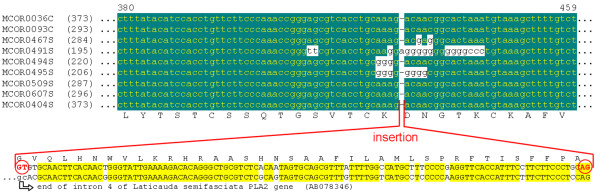
**Segment of PLA2 clusters, showing the presence of a 114 bp insertion in MCOR0404S**. This inserted sequence is very similar to the last 114 bp of the fourth intron of PLA2 gene from several snakes, including *L. semifasciata *(aligned above the insertion), and is delimited by the canonic GT and AG in intron/exon junctions.

It was interesting to note that the singlet cluster MCOR0404S, shows a peculiar sequence due to a 114-bp insertion in position 110, within the mature protein (Figure 3). Comparing the sequence of this insertion against databases, it is possible to see that it corresponds to the last 114 bp of the fourth intron of some elapidic PLA_2 _genes, such as that from *Laticauda semifasciata *[23]. These introns 4 of PLA_2_s usually have around 500 bp, but only 114 bp are present in the insertion of MCOR0404S cDNA. Assuming a conserved gene structure of PLA_2 _genes among species, this insertion may represent possible incomplete splicing of the transcript, since a 3' portion of the intron remained attached to the mRNA. In fact, the insertion starts with the dinucleotide GT and ends with AG, precisely the canonic sequence for intron/exon junctions (Figure 3). Besides, the insertion of 114 bp is a multiple of 3, and hence, it corresponds to 38 codons (without stop codons). This allows, after the insertion, the continuity of the reading frame, keeping the original C-terminal region. Although this insertion could originate from a random mistake in the splicing process, the absence of stop codons or cysteine residues (which would destabilize the structure) and the fact that it keeps the translational phase are indicative that it could result in a new protein product. Nevertheless, the analysis of the inserted amino acid sequence does not display similarity with any known domain. This is another example of indels frequently observed in toxin cDNAs [24].

#### C-type Lectins

C-type lectins are non-enzymatic proteins present in the venom of snakes of all families. Like plant lectins, some of the venom lectins are able to bind to carbohydrates. In the presence of Ca^2+^, the C-type lectins begin several biological processes, such as adhesion, agglutination, endocytosis and neutralization of the pathogen. The lectins could be divided into seven different groups according to their structural characteristics [25]. The C-type lectins that have lost their capability of binding to carbohydrates are called lectin-like, because they still maintain the structural characteristics in common with true lectins. Venom lectins may act as agonists or sometimes antagonists in platelet aggregation and affect thrombosis and homeostasis by activating and inhibiting specific receptors in platelet membranes [26].

The C-type lectins found here were also abundant toxins. Sixty-five clones show similarity with these proteins and were grouped into 13 clusters, representing approximately 5% of the total transcripts expressed in the tissue. Many of the clusters correspond to full-lengths ORFs that could be aligned with other C-type lectins from venom [see Additional file 2].

C-type lectins are ubiquitous components of the venom, but are very diversified in structure-function terms, and thus, it is interesting to understand how the C-type lectins from *M. corallinus *fit in the phylogeny of lectins. A Bayesian phylogenetic analysis revealed that the proteins of snake venom set in a unique group, distinct from the animal physiologic lectins [see Additional file 2]. Within the group of venom lectins, it is possible to see a clear distinction between the true C-type lectins (carbohydrate-binding) and their structural homologs, the lectin-like (probabilities 0.81 and 1.0, respectively). All the *M. corallinus *lectins are arranged inside the true C-type lectin group, together with the other elapids lectins. The only exception is the cluster MCOR0067C (clearly distinct from the others in alignment), which is more similar, in terms of identity, to the short physiologic proteins related to lithostatin than to the venom lectins (true or not). Recently, the same kind of sequence was observed in the *B. insularis *transcriptome (BINS0004C). Thus, the finding of another lectin with those characteristics supports the possibility that this lectin could be a third group of venom lectins or that it has a specialized function in the venom gland.

For the organization inside the branch of true lectins, the majority of clusters are grouped together with the lectin of *Lapemis hardwick*, an elapid of the *Hydrophiinae *subfamily (sea snakes). Only one cluster, MCOR0090C, is grouped with the real lectins of other elapids and viperids. Either way, these components seem to be well diversified in *M. corallinus*.

#### Natriuretic Peptide (NP)

NPs have been described in snake venoms, where their precursors are organized in different ways. In the Elapidae, a 110- to 140-amino acid ORF contains a signal peptide, a short propeptide and the NP with a C-terminal extension resembling that of vertebrate atrial and brain NPs (A/BNPs) [27]. In contrast, in the Viperidae (*Crotalinae *subfamily), the precursor is a more complex molecule with a 180- to 270-amino acid ORF containing a signal peptide, a region with a variable number of the pharmacologically relevant bradykinin-potentiating peptides (BPPs) (from one to seven in different snake species) followed by a long intervening linker sequence and the C-type NP (CNP), without a C-terminal extension [28]. In the venom of the dipsadid snake *Philodryas olfersii*, it is similar to the Viperidae BPP/CNP precursor, including the signal peptide, the linker and the CNP (without C-terminal extension), but lacking the entire BPPs region [9]. The BPPs are important inhibitors of the angiotensin-converting enzyme that served as basis for the design of the anti-hypertensive drug captopril.

Here we found 5 clusters, totaling 14 clones. The cDNA representing the cluster MCOR0001C (6 clones) is very similar to the one previously characterized [27], except for the absence of the extra cysteine observed in that cDNA. In fact, resequencing the original clone from Ho and colleagues, it was clear that the sequence described earlier was incorrect (data not shown), and that the correct one corresponds to that of the MCOR0001C cluster. All the natriuretic peptides described represent a structural ring with 17 amino acids formed by an expanding disulfide bond with a few amino acids in the N-terminal. In the C-terminal region, the type B and C of mammalian NPs have 4 to 7 amino acids. The natriuretic peptide of *M. corallinus *has some peculiarities: a) the peptide has an unusually longer C-terminal extension; and b) this possible natriuretic peptide is flanked by 2 homologous regions with unknown function [27].

**Figure 4:**
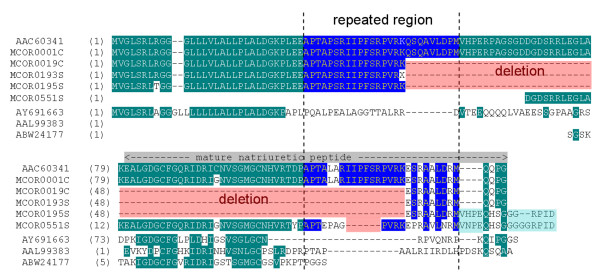
**Alignment of the deduced amino acid sequence of full-length NP precursor clusters (MCORs) and other NP precursors, including the previously described *M. corallinus *AAC60341**. A repeated region is shown between dashed bars, with the conserved residues in blue. Some "in frame" deletions and insertions, with regard to the previously described AAC60341, are shaded in red and light blue, respectively. The predicted region of mature natriuretic peptide is indicated above the alignment.

Besides this archetypal type, three other clusters (MCOR019C, MCOR0193S and MCOR0195S) show a deletion of 237 bp relative to MCOR0001C, resulting in the absence of 79 amino acids (Figure 4). The reading frame is maintained after the deletion and the resulting polypeptide has a signal peptide, part of the pro-domain, including one part of the repeated region, and the rest of the C-terminal. Therefore, the deleted section corresponds to mature NP and part of the flanking region. Unlike the insertion in the cDNA of PLA_2 _(Figure 3), the deleted section does not follow the GT-AG rule of splicing and, therefore, does not seem to be derived from an alternative splicing. Besides that, some clusters (MCOR0195S and MCOR0551S) still show insertions corresponding to four residues (VH/NPE) next to the C-terminal end.

It is difficult to determine the importance of such molecule that is abundant, with part of the pro-domain and the C-terminal end, but not showing the NP. One clue could be the presence, even in the deleted form, of one of the repetitive regions, which has been suggested to have typical elements of active biological peptides [27]. This precursor may be chosen to produce a new peptide based on this segment. It is interesting to note that the BPPs/CNPs precursors, whose structures have been known for a while, are now revealing new active peptides in their pro-peptide regions. Graham and colleagues, in 2005 [29], found bradykinin inhibitory peptides in the same precursor of the BPPs. Recently, Wagstaff and colleagues described that the conserved spacing region from some precursors generates peptides (EKW) capable of inhibiting the metalloprotease in the same venom, in a supposed mechanism of protection against proteolysis in the gland [30].

#### L-Amino Acid Oxidase

These are larger enzymes of 58 kDa and act basically on L-amino acids, converting them to keto acids and generating H_2_O_2_, which seems to contribute to the inhibition of platelet aggregation and to cause cellular injury [31]. It was not possible to obtain a full-length clone from our dataset, probably due to the very long cDNA (2.8 kb) [32]. Two clusters, MCOR0200S and MCOR0263S, each one containing just one clone (singlet), were found in the *M. corallinus *databank. L-amino acid oxidase has already been reported in elapids (ex.: AAY89682), but not in *Micrurus*.

#### Kunitz Inhibitors

In snake venoms, the scaffold of Kunitz-type serine protease inhibitors occurs as two functionally distinct molecules: the non-neurotoxic serine protease inhibitor, and the neurotoxic ones, also called dendrotoxins, lacking major inhibitory action on proteases. The former ones are capable of inhibiting trypsin and chymotrypsin, being common in both the Viperidae and Elapidae [33]. The dendrotoxins are present in the venom of some elapids [34], such as the black mamba (*Dendroaspis*) and the kraits (*Bungarus*). They exert their toxic action by increasing the release of neurotransmitters, such as acetylcholine, from pre-synaptic membranes of cholinergic synapses. The specific targets of dendrotoxins are high-voltage-activated Ca^2+ ^channels (L-, N-, and P-type) in neuronal cell membranes, which are blocked [35].

Two low expressed clusters, MCOR0110C and MCOR0611S, were found in this database. Both seem to be more similar to the inhibitors than to the neurotoxins, matching several Viperidae inhibitors, although the degree of conservation between these two types is low, and further analysis is needed in order to define their exact function.

#### Metalloproteases

Venom metalloproteases are normally grouped according to the presence of a specific domain. All of them have a proteolytic domain characterized by the presence of a catalytic site conserving the motif HEXXHXXGXXH, responsible for the binding of a metal ion, generally Zn^2+^. After the catalytic domain, there may be a disintegrin domain, capable of binding to platelet integrins or to endothelial cells. This region could contain the RGD motif (RGD disintegrins) or have its sequence changed to another, such as ECD (non-RGD disintegrins), which would change its specificity. The C-terminal region of non-RGD disintegrins could also have a cysteine-rich domain with unknown function. These enzymes are very abundant in the family Viperidae but are not so common and diversified in the Elapidae.

The metalloprotease found here is represented by one partial cluster, MCOR0063C, containing just 4 clones (1123 bp). It is similar to cobrin found in *Naja naja*, a species of the family Elapidae from the Old World. It is an example of a P-III type of metalloprotease, i.e., the sequence shows a non-RGD disintegrin type followed by a cysteine-rich domain [36].

#### Serine Proteases

Snake venom serine proteases act on elements of the coagulation cascade and are believed to occur as trypsin-like enzymes, widely described in the Viperidae, or as the more complex factor X-like proteins found in some terrestrial Elapidae [37]. Recently, some trypsin-like enzymes were described in the Elapidae, e.g., ABN72544, showing that the factor X-like is not the only serine protease fold in this family.

In the transcriptome of *M. corallinus*, we did not find either type, but only a partial singlet cluster (MCOR0160S), showing more similarity to non-venom serine proteases from mammals (~52% identity), than to snake ones (~39%). The main hits are with prostasins from basal mammals such as platypus (*Monotremata*) and opossum (*Metatheria*). This is a channel-activating protease involved in the growth of epithelial cells. The portion matching MCOR0160S corresponds to a part of the peptidase S1 domain, indicating that proteolytic activity should be present. Since the sequence is partial, it is not possible to define the presence of extra domains, such as the transmembrane or the propeptide C-terminal end. Due to the ubiquitous presence of serine protease in snake venoms, we include this one among the possible toxins from *M. corallinus*, although further confirmation is still needed.

#### NGF

Like their homologs in mammals, responsible for regulating neuronal differentiation, NGFs (neurotrophic factor family) of venom act as low potential agonists of TrkA receptors (tyrosine-kinase), competing with endogenous NGF in binding to the receptor and influencing the development of cholinergic neurons. Besides inducing a typical growth of fibers in cell culture, snake venom and human NGFs also exhibit non-neuronal effects, such as the induction of plasma overflow or the release of histamines from blood cells [38]. The effect of these factors in envenoming probably renders the bite site more susceptible to other components of the venom and distribute the substances that are difficult to infiltrate the target tissue [39].

This component was also found in our library, although less abundant than in other transcriptomes. Probably due to the length of the sequence, the only cluster obtained (MCOR0149S) is partial.

#### WAP

WAP proteins (whey acidic proteins), or waprin or nawaprin (described first in *Naja nigrocollis*), play a possible role as a protease inhibitor. It is similar to elafin (elastase inhibitor of human leukocytes), clatrin-like (calcium transport inhibitor) and other extra cellular protease inhibitors [40]. The WAP domain generally consists of 50 amino acid residues, with 8 conserved cysteine residues forming 4 disulfide bridges. Although the cysteines residues are conserved, the inter-cysteine segments are totally different from those of other WAP family members. Among the ESTs of *M. corallinus*, we obtained only one cluster, MCOR0526S, containing just one clone coding for a WAP protein. It is a partial sequence without signal peptide and part of the mature protein.

### General considerations on *M. corallinus *transcriptome

Whereas other snake families have been well investigated through the transcriptomic approach, Elapidae cDNA sequences were obtained only from timely cloning efforts. The toxins in the *M. corallinus *transcriptome presented here represent approximately 46% (625 clones grouped in 93 clusters) of all the information. This very high expression of a functionally related group of protein is expected since this tissue is specialized in toxin synthesis, and is similar to the results observed from other snake families such as the Viperidae [5-7] and Colubridae [9].

The general profile of toxins revealed a complex diversity of toxins (10 different classes of toxins). Protein profiles of *Micrurus *venoms (e.g., [10,41]) usually show a large number of proteins of around 6–24 kDa (mainly 3FTx with various activities and PLA_2_) with minor bands at higher molecular mass. Although PLA_2 _and 3FTx make up the vast majority of ESTs, 8 other classes were found, including proteins with a predicted higher molecular mass. All these classes have been previously observed in at least one species of the Elapidae, but as far as we know, not in the same species or genus. Olamendi-Portugal and colleagues [41] investigated the proteome set of a Central America coral snake (*Micrurus surinamensis*) and found that the main group of toxins corresponded to 3FTx. However, they also identified PLA_2_, other low-molecular mass proteins and L-amino acid oxidase. Together, these data suggest that the complexity of venom components in *Micrurus *is readily comparable with that of the family Viperidae. Of course, other datasets are needed to extrapolate these *Micrurus *profiles.

Nevertheless, the neurotoxic action of pre- and post-synaptic toxins still predominate. Considering this action, *M. corallinus *venom is frequently compared and contrasted with that of *M. frontalis*, because both are the major species in terms of occurrence and accidents in Brazil, but the latter shows an almost exclusively post-synaptic venom, whereas the former has both pharmacological actions [2]. The presence of elevated levels of PLA_2 _messengers in *M. corallinus*, almost equal to the 3FTx levels, is in agreement with this, suggesting that the responsibility for this action may be associated with the level of this component. Unfortunately, we still do not have the *M. frontallis *transcriptome data in order to compare them.

Besides the existence of diverse classes of toxins, it is worth noting the large number of different cDNAs in each class. 3FTx, for example, are represented by 52 differently assembled cDNAs. It is true that many forms may result from the assembly of non-overlapping segments or correspond to cDNAs with few nucleotide polymorphisms (with many silent substitutions), which may be associated with individual variation resulting from the pool of the venom glands used. Nevertheless, exclusively considering the amino acid sequences from the full-length 3FTx, 10 of the 28 full-length 3FTx proteins observed are less than 80% conserved relative to any other, and 7 of them are less than 50% conserved relative to any other. If we exclude the signal peptide from the analysis, percentages are obtained that reflect even more diversity. This indicates the great variability of these sequences in a single species. It is surprising that a species with a very specialized diet (feeding mainly on other small snakes) shows this diversity. However, in the proteome of M. *surinamensi *various subtypes of 3FTx are present. Five of the most abundant, including short- and long-chain 3FTx, were functionally characterized and shown to be acetylcholine receptor with different affinities and peculiar specificities. *M. surinamensi *is also a diet specialist, feeding on fish, but many of their toxins seem to be active also in mammals [41]. In a family of short toxins subjected to high evolutionary pressures on structure-function relationships, this may represent very different kinds of activities, such as several receptor specificities.

### Identification of putative antigenic candidates

Considering the problems in the production of *M. corallinus *antiserum for humans due to the difficulties in obtaining its venom, alternative ways to obtain their antigens are important. The direct use of *M. corallinus *recombinant proteins as immunogens have been tested before [10,11] and although they were shown to produce immune responses indicating the recognition of the native proteins, very complicated steps were required for protein refolding and to obtain active toxins. Therefore, we decided to evaluate the feasibility of using cDNAs derived from this transcriptome initiative as immunogens for producing an antiserum. By analyzing the dataset, we confirmed the predominant toxins in this venom and suggested some proteins that could represent good antigenic candidates.

The proposed candidates should code for proteins that are abundant in the venom. Since 3FTx and PLA_2 _account for 85% of toxin ESTs, they were chosen for this first evaluation of immune response through DNA immunization. The 3FTx candidates were selected based on the abundance of each transcript, with aim of choosing the toxins that would be abundant in the venom. Among them, we considered only the most divergent forms, indicated by a neighbor-joining tree, avoiding the selection of closely related isoforms. Thus, we aimed to select diverse abundant (potentially important) cDNAs of 3FTx, which are the major toxins, and one representative of PLA_2_, rather than to choose the most immunogenic ones, as used elsewhere [14].

The 35 clusters of 3FTx with full length sequences were aligned and self-compared, giving rise to groups of clusters showing more similarity (Figure 2) [see Additional file 1]. One group containing 5 clusters and 45 clones was represented by MCOR0604C, here named Atg1, and is similar to the nxh8 (AJ344067). The second group (Atg2) is the most abundant 3FTx set, containing 117 clones, represented by the MCOR0599C cluster, which is similar to the neurotoxin homologs 7/3/1 (nxh1, nxh3, nxh7). Among the remaining 3FTx, two subgroups were defined: Atg3 included 29 clones (represented by the MCOR0039C cluster) and Atg4 had 27 clones (represented by the MCOR0100C cluster).

The obvious fifth candidate (Atg5) was the MCOR0036C cluster, representing the most abundant cluster obtained (196 clones), corresponding to the putative *M. corallinus *PLA_2_.

### DNA immunization and immune response

The five selected candidates were subcloned in the mammalian vector, pSecTag2A and the DNA injected in mice. In parallel, some constructions of the same cDNAs cloned in prokaryotic expression systems were used to produce recombinant antigens (data not shown) for the evaluation of the antisera.

**Figure 5:**
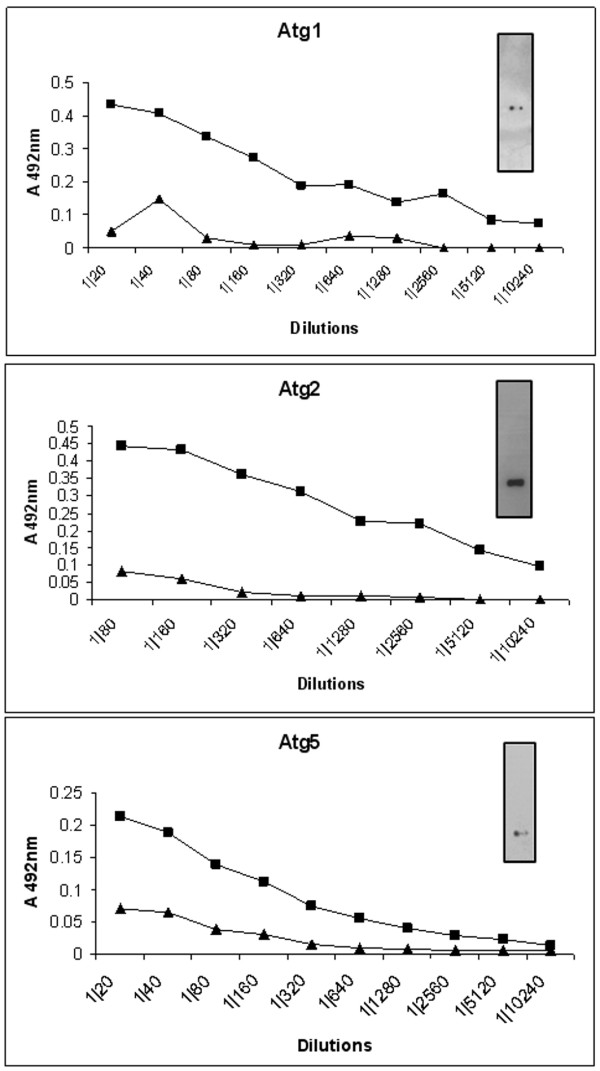
**Induction of immune response in BALB/c mice immunized with DNA vaccine**. Total IgG against the selected antigens (DNA from Atg1, Atg2 or Atg5) were detected through ELISA in plates coated with the respective recombinant protein. Lines with black triangles and black squares indicate, respectively, pre-immune and final sera (two weeks after the last immunization). Each point represents an average of three different measures of the same immunization. Inserts show the detections of the same proteins by western-blot.

In order to determine if the DNA immunizations would be capable of generating an immunological response to the proposed 3FTx candidates, ELISA tests were performed using the anti-sera raised from DNA immunization with Atg1 and Atg2 to recognize the respective recombinant proteins. The results are presented in Figure 5, where it is possible to see a much stronger recognition of the recombinant proteins by the immune sera than by the pre-immune one. The highest titers were obtained for Atg2 (1:10240) and then Atg1 (1:5120). These results indicate that there were differences between the production of antibodies in all three candidates. Besides the ELISA tests, immunodetection by Western blotting was performed (inserts in Figure 5). In this case, the recombinant proteins could also be recognized by their respective sera, Atg1 and Atg2. The two tests indicate that the 3Ftx candidates produced antibody titers with DNA immunization in mice. However, the amplitudes of the responses were quite low, reaching optical density values ranging from 0.25 to 0.5.

To evaluate the antiserum raised against the two other 3FTx antigens, ELISA plates were coated with the 3FTx recombinant proteins available (Atg1 and Atg2) and exposed to the sera obtained from Atg3 and Atg4 DNA immunization. Therefore, a positive response would indicate that there is cross reaction between the selected antigenic candidates. In this test, the sera of mice immunized with Atg3 or Atg4 showed better recognition of the recombinant proteins corresponding to Atg2 than those corresponding to the Atg1 [see Additional file 3]. This indicates that Atg1 has less conserved epitopes with the other antigens analyzed, which is in accordance with its sequence properties that showed MCOR0604C (nxh8) as a truly distinctive type of 3FTx. Besides, the low cross-reaction titers even with the most conserved Atg2 suggests that the distinct 3FTx isoforms selected, alone, may not elicit a response sufficient to neutralize the venom. For this reason, a set of these toxins should be used together to ensure complete protection.

The immunological response against Atg5 (PLA_2_) only showed low reaction titers (1:160), but was also capable of recognizing the recombinant proteins in Western blots (Figure 5).

**Figure 6:**
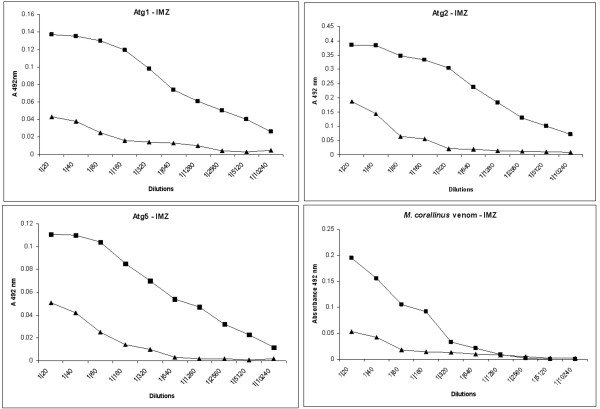
**Induction of immune response in BALB/c mice co-immunized with DNA vaccine**. Total IgG against a pool of selected antigens (DNA from Atg1, Atg2 and Atg5) were detected through ELISA in plates coated with each of three recombinant proteins or the venom, two weeks after the last immunization. Lines with black triangles and black squares indicate, respectively, pre-immune and final sera (two weeks after the last immunization).

Atg1, Atg2 and Atg5 DNA were then co-administered in mice and the serum tested against the respective recombinant proteins and against crude venom from *M. corallinus *(Figure 6). The best recognition was seen with Atg2 (1:5120), followed by Atg1 (1:320), Atg5 (1:120) and *M. corallinus *venom (1:120).

Therefore, our results indicate that immunization with DNA is able to generate an immunological response against recombinant proteins. Meanwhile, the venom was poorly recognized by these sera in ELISA. Perhaps, optimizing the immunization strategy would improve the recognition. For practical reasons, in this first evaluation we utilized here a simple *intra-muscular *protocol, by injecting the DNA in mice in their anterior tibia muscle or quadriceps. Epidermal injection should improve the immune response, as previously demonstrated [42], particularly if associated with biobalistic protocols. This procedure has already been shown in several studies to be the most efficient to induce an immunological response of the Th2 type (IL-4, IL-5, IL10), efficient to generate more antibodies, which is desirable for hyperimmune serum production. Nevertheless, the reaction titers of anti-toxin sera are not directly related to its efficiency; if some important epitopes were neutralized, the general toxicity of the venom could be blocked even with low-titer serum, as clearly shown before [14].

Years of DNA immunization studies have shown that, unlike with inactivated antigen vaccines or subunit vaccines, genetic vaccines result in an antigenic presentation via MHC class I and MHC class II molecules, which mimics the resulting process of natural infection to activate T lymphocytes CD4+, CD8+ and the production of antibodies. The different types of induced immunological response for DNA administration clearly justify their application in the infectious disease field [11]. The question is how to derive this specific answer for the purpose of generating a hyperimmune serum, and less cellular protection. Some attempts to achieve this objective are in progress. Besides the few DNA immunization works referring to snake toxins, some efforts are being made to investigate the generation of hyperimmune serum against bacterial or viral diseases. Fischer and colleagues in 2003 [43], for example, using a cationic lipid composition together with DNA, generated reasonable titers of antibodies against rabies virus in horses. Herrmann and colleagues in 2006 [44] were capable to produce an antiserum in rabbits against anthrax that was capable of neutralizing the deleterious effects of the *Bacillus anthracis *toxin. Thus, quoting Harrison et al., 2000, [42]: "The application of DNA-based methodologies to the development of therapeutic antivenom represents the first major conceptual change in antivenom production in over a century and has the potential to provide a more cost-effective, less hazardous and more immunologically specific therapy than those used currently to treat envenoming by snakes"

## Conclusion

The transcriptome analysis of *M. corallinus *provides a large profile of Elapidae toxin cDNAs. Ten classes of possible toxins were found, representing a great diversity of toxins for a venom believed to be almost exclusively neurotoxic. Nevertheless, the possible neurotoxins (3FTx and PLA_2_) are in fact the majority, totaling 85% of toxin transcripts. The possible post-synaptic components (3FTx) are very diverse in terms of sequences, possibly aiming to achieve different kinds of receptors. The pre-synaptic component (PLA_2_), in contrast, is more conserved, with the main transcript being represented by 196 of the 1438 ESTs analyzed here. Nevertheless, the high expression of both types of possible neurotoxins is in agreement with the known presence of pre- and post-synaptic activities in the venom of this species.

From a biotechnological point of view, this transcriptome set represents a library of naturally selected templates, especially for molecules acting on nicotinic receptors or ion channels which may be useful for pharmacological purposes. Regarding envenomation treatment, the utilization of genetic immunization based on the survey of transcriptome data carried out here was shown to be feasible for generating immune responses, although more optimization is still needed. Although far from therapeutic application, these findings represent the first steps in the production of an alternative anti-*M. corallinus *venom serum.

## Methods

### cDNA library preparation

Two cDNA libraries were constructed in 1992, with *RNA poli(A+) *extracted from 10 specimens of *M. corallinus *[27]. The cDNAs were divided in two fractions (400 to 600 bp and >600 bp) and the transcripts were linked to λ*gt11D *(Pharmacia, USA) pre-digested phage with EcoRI/NotI restriction enzymes. The phage library was amplified after infection in *E. coli *Y1088.

To isolate bacteriophage plaques, first a colony of *E. coli Y1088 *was inoculated in 5 mL of NZCYM culture media containing 0.2% of maltose [45]. After the addiction of 0.01 M MgSO_4_, the absorbance was accomplished until reaching 2.0 and then kept at 4°C. Dilutions of phage libraries were mixed with 100 μL of this *E. coli Y1088 *preparation and incubated for 20 minutes at 37°C. NZCYM culture media containing top agarose (0.7% agarose) was then added to the mixture and it was distributed over a bottom agar (1.5% agar) plate prepared with NZCYM culture media. After 12–16 hours at 37°C, the phage plaques were ready to be isolated.

### Amplification of phage cDNA insert by PCR and DNA sequencing

Each lytic colony was collected using a Pasteur pipette tip and inserted into a microcentrifuge tube containing 200 μl of SM media [45]. After 1 hour of incubation with agitation at room temperature, 10 μl of this solution was used to amplify the cDNA inserted in the genetic material of each phage with Taq polymerase enzyme (Platinum Taq DNA Polymerase – Invitrogen) and λgt11F and R primers by PCR, in a 96 well plate. Eight microliters of the amplified product was then chemically cleaned by the addition of 5 units of Exonuclease I (GE Healthcare), 1 unit of Shrimp Alkaline Phosphatase (SAP) (GE Healthcare) for 1 hour at 37°C and then inactivated for 15 minutes at 80°C. From the purified PCR product, 300 ng was sequenced with BigDye2 dideoxy-terminators (Applied Biosystems) and λgt11F primer on an ABI 3100 sequencer.

### Cluster assembly and identification

After sequencing the DNA, the electropherogram files were analyzed in a semi-automatic way, as described [5]. The Phred program was used  to remove bad quality sequences (window length of 75 bases with 75% of standard quality < 25). Then, adapter and phage sequences were removed by the CrossMatch Program. An examination was carried out manually and sequences below 150 bp were discarded. ESTs were then assembled in clusters of contiguous sequences using the CAP3 program [46], set for 98% or more of base identity in a high-quality region. The cluster sequences were searched in the GeneBank NCBI database with BLASTX and BLASTN algorithms. Non-identified sequences and those with unpredicted function were checked for the presence of signal peptide by SignalP 3.0 program . A final annotation table was generated in Microsoft Excel format containing all the relevant information about clusters. ESTs sequences were deposited in Genbank dbEST under accession numbers FL589790 to FL591230.

### Antigenic candidates selection, cloning and immunization

Five cDNAs were selected after an analysis of the *Micrurus corallinus *transcriptomic databank and named Atg1 to Atg5 (Antigens 1 to 5), as described in the Results. Specific oligos were drawn for each antigenic candidate. The mature protein region was amplified by PCR and cloned, first, in pGEM-T vector, then digested and subcloned in the eukaryotic expression vector, pSecTag2A (Invitrogen). This vector allows the secretion of the protein to the medium due to the secretion signal from the kappa chain of immunoglobulins. The expressed protein also contains some tags like a poly-histidine (His) and the myc epitopes (c-myc). To obtain the large amount of DNA needed for the immunization process, maxipreps were made with the Qiagen Plasmid Purification kit (Qiagen). They were then quantified and its purity was determined (OD 260/280) by spectrophotometry.

The DNA immunization of the candidates was made in five groups of BALB/c mice, females of homogenous weight (18–22 g) and age (6–8 weeks old). The experiments in animals was approved by the Ethics Committee of Butantan Institute, under protocol 258/06, according to international guidelines. Each group of five animals was immunized with an antigenic candidate. The animals were anesthetized i.p. with approximately 200 μL of anesthetic (ketamine 500 μL/10 mL and xilazine 1000 μL/10 mL). The injection of the DNA vaccine was intramuscular (IM). Each animal received 100 μg of DNA *per dose *and one dose every 15 days for a total of three doses (300 μg/animal). The same procedure was used to immunize a group of five animals with a pool of three immunogens (33 μg of Atg1, 33 μg of Atg2 and 33 μg of Atg5 *per dose*).

### Immune response analysis

Mice were bled individually from the retro-orbital plexus to detect antibodies in the blood through ELISA (Enzyme-linked Immunosorbent Assay). An initial bleeding was done before the immunizations, in order to obtain the pre-immune serum and the final bleeding was made after the third dose. Recombinant proteins from 3 of the 5 candidates (Atg1, Atg2, Atg5) were produced in *E. coli*, as His tagged fusion proteins, and purified by Ni^2+ ^affinity chromatography, as described previously [10,11]. These proteins (1 μg) were used for the ELISA coating in NaHCO_3 _and Na_2_CO_3 _0,05 M pH 9,6 buffer. After blocking it with 10% milk in PBS, dilutions of the sera were prepared in ranges from 1:20 to 1:10240 and incubated during 1.5 hour with a 1% BSA + 0.1% PBS-T solution, in 37°C. The plates were then incubated with a secondary antibody, IgG anti-mouse conjugated with peroxidase for 1 hour in 37°C and revealed with 100 μl of 0.4 mg/ml OPD (ortofenilenodiamine), 0.05% H_2_O_2 _in citrate-phosphate buffer (0.1 M citric acid + 0.2 M sodium phosphate). The reaction was blocked with the addition of 50 μl of 8N H_2_SO_4 _after 15 minutes. The experiments were realized in triplicates.

## Authors' contributions

LIL performed all the bench work on EST generation and immunology, provided the first annotation and drafted the manuscript. PLH constructed the cDNA library, conceived the study and discussed the results. ILMJA coordinated the study, obtained the funding, performed the bioinformatics, reviewed the annotation and drafted the manuscript. All authors read and approved the final manuscript.

## Supplementary Material

Additional file 1 **The charts show the relative proportions of each 3FTx cluster with their number of ESTs.** Click here for file

Additional file 2 **An alignment and a phylogenetic analysis of C-type lectins.** Click here for file

Additional file 3 **ELISA results showing the detection of Atg1 and Atg2 with sera raised against Atg3 and Atg4.** Click here for file
