# Supplementary material for: Transcriptomic basis for an antiserum against Micrurus corallinus (coral snake) venom
Source: BMC Genomics. 2009 Mar 16;10:112. doi: 10.1186/1471-2164-10-112 (PMC2662881; doi:10.1186/1471-2164-10-112)
Supplement: Additional file 1 — The charts show the relative proportions of each 3FTx cluster with their number of ESTs. [file 1471-2164-10-112-S1.pdf]

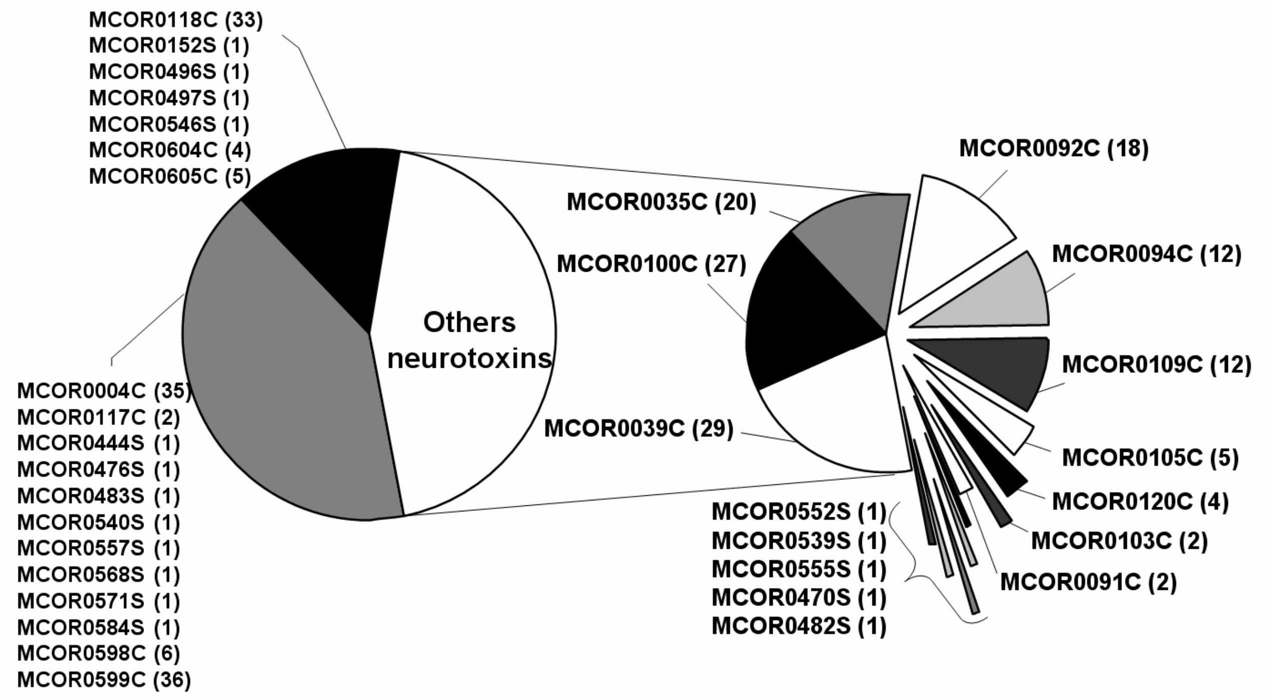

Additional file 1: The diversity of 3FTx clusters found in *M. corallinus* venom gland transcriptome. In parentheses are the number of ESTs in each cluster.
