# Supplementary figures and images for: Transcriptomic basis for an antiserum against Micrurus corallinus (coral snake) venom
Source: BMC Genomics. 2009 Mar 16;10:112. doi: 10.1186/1471-2164-10-112 (PMC2662881; doi:10.1186/1471-2164-10-112)

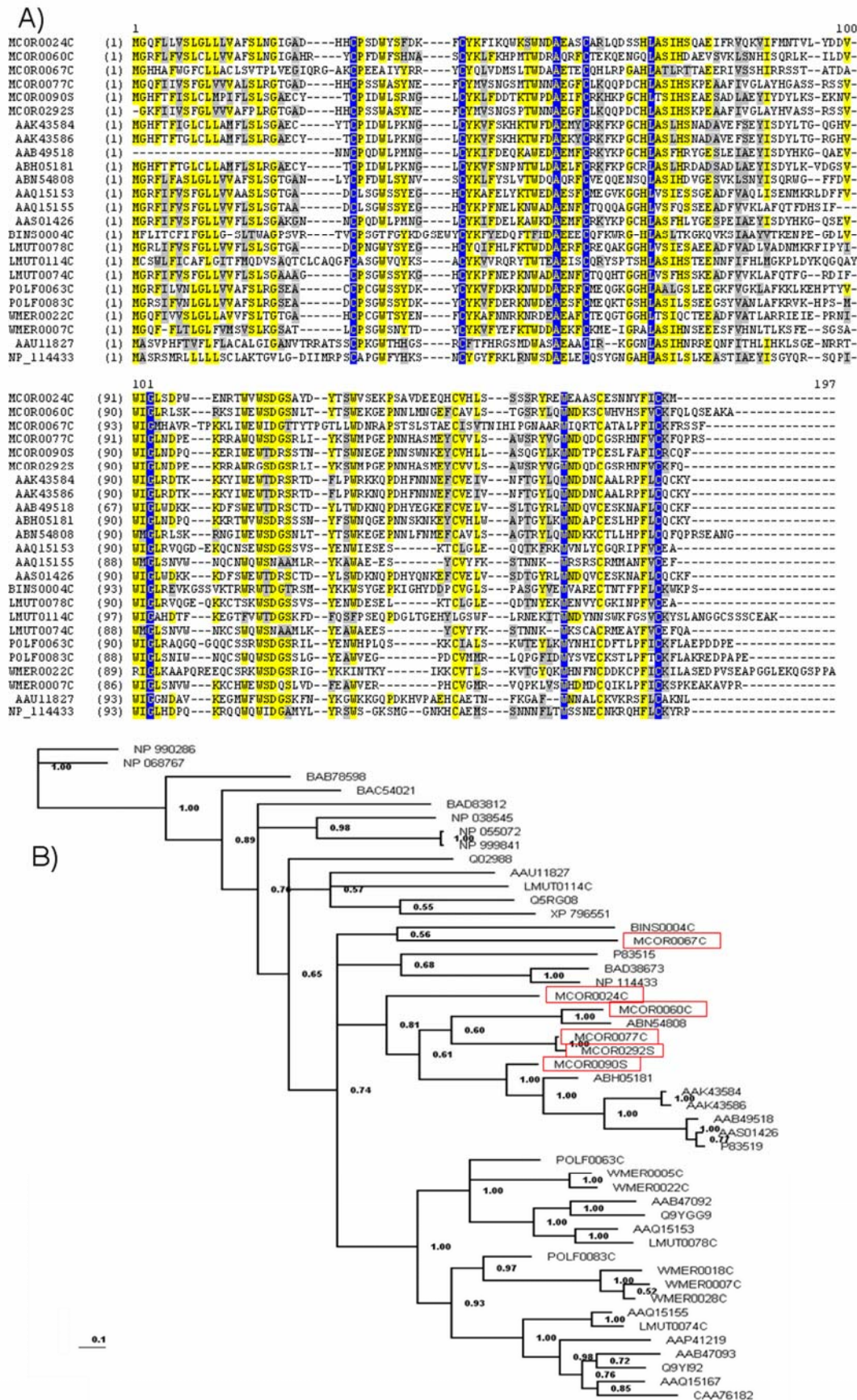

Supplement: Additional file 2 — An alignment and a phylogenetic analysis of C-type lectins. [file 1471-2164-10-112-S2.pdf]
