# Supplementary material for: Transcriptomic basis for an antiserum against Micrurus corallinus (coral snake) venom
Source: BMC Genomics. 2009 Mar 16;10:112. doi: 10.1186/1471-2164-10-112 (PMC2662881; doi:10.1186/1471-2164-10-112)
Supplement: Additional file 3 — ELISA results showing the detection of Atg1 and Atg2 with sera raised against Atg3 and Atg4. [file 1471-2164-10-112-S3.pdf]

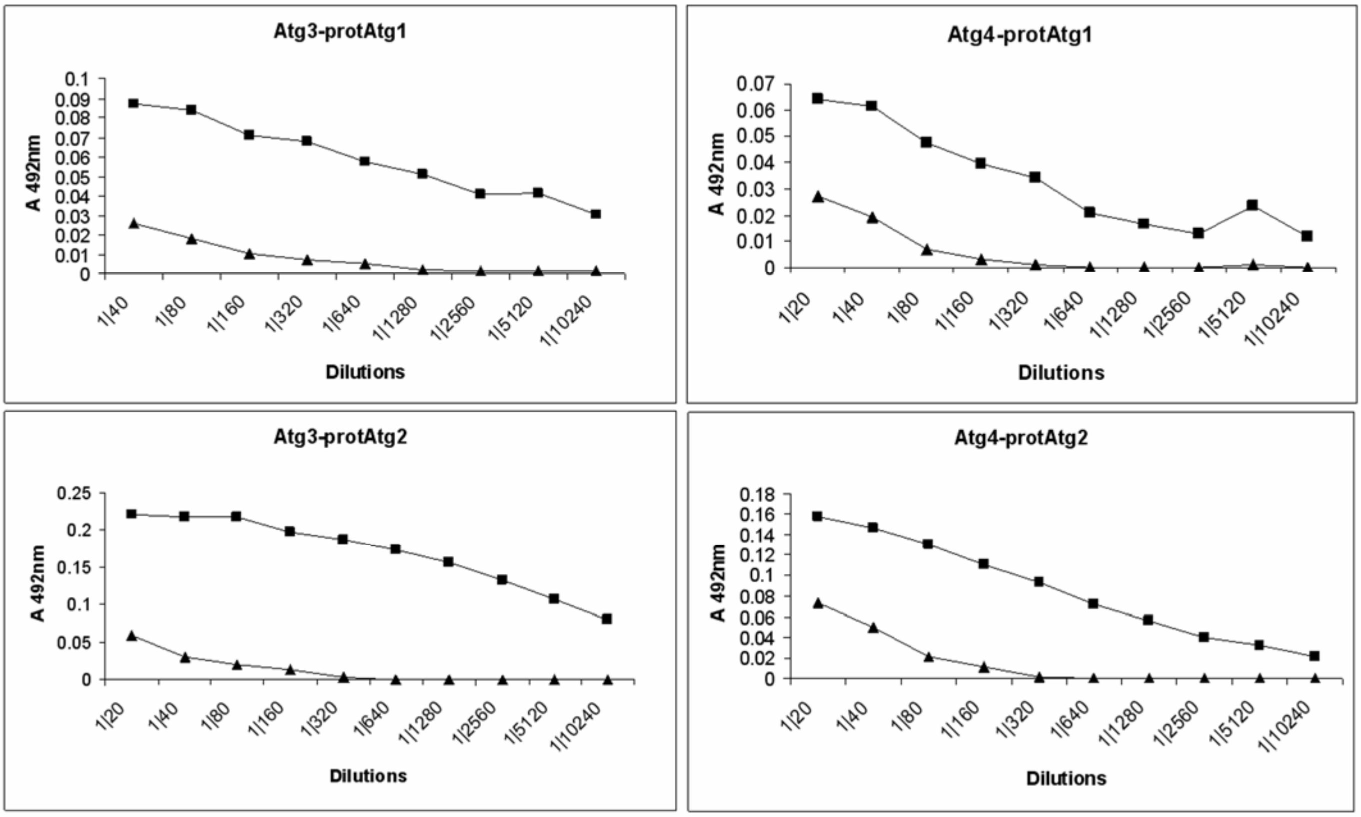

Additional file 3: Cross-detection of immune response in BALB/c mice immunized with DNA. Total IgG against the antigens Atg3 (left) and Atg4 (right) were cross-detected through ELISA in plates coated with recombinant proteins Atg1 and Atg2. Lines with black triangles and black squares indicate, respectively, pre-immune and final sera (two weeks after the last immunization). Each point represents an average of three different measures of the same immunization.
